# A Machine-learning Approach for the Assessment of the Proliferative Compartment of Solid Tumors on Hematoxylin-Eosin-Stained Sections

**DOI:** 10.3390/cancers12051344

**Published:** 2020-05-25

**Authors:** Francesco Martino, Silvia Varricchio, Daniela Russo, Francesco Merolla, Gennaro Ilardi, Massimo Mascolo, Giovanni Orabona dell’Aversana, Luigi Califano, Guglielmo Toscano, Giuseppe De Pietro, Maria Frucci, Nadia Brancati, Filippo Fraggetta, Stefania Staibano

**Affiliations:** 1 Department of Advanced Biomedical Sciences, Pathology Unit, University of Naples Federico II, 80131 Naples, Italy; francesco.martino@unina.it (F.M.); silvia.varricchio@gmail.com (S.V.); gennaro.ilardi@unina.it (G.I.); massimo.mascolo@unina.it (M.M.); staibano@unina.it (S.S.); 2 Department of Medicine and Health Sciences “V. Tiberio”, University of Molise, 86100 Campobasso, Italy; 3 Department of Maxillofacial Surgery, University of Naples “Federico II”, 80131 Naples, Italy; giovanni.dellaversanaorabona@unina.it (G.O.d’A.); califano@unina.it (L.C.); 4 Healthcare Informatics Services, A.O.U. Federico II, 80131 Naples, Italy; toscano@unina.it; 5 Institute for High Performance Computing and Networking of National Research Council of Italy, ICAR-CNR, 80131 Naples, Italy; giuseppe.depietro@icar.cnr.it (G.D.P.); maria.frucci@cnr.it (M.F.); nadia.brancati@icar.cnr.it (N.B.); 6 Pathology Unit, Azienda Ospedaliera per l’Emergenza Cannizzaro Hospital, 95126 Catania, Italy; filippofra@hotmail.com

**Keywords:** Ki67, digital pathology, machine learning

## Abstract

We introduce a machine learning-based analysis to predict the immunohistochemical (IHC) labeling index for the cell proliferation marker Ki67/MIB1 on cancer tissues based on morphometrical features extracted from hematoxylin and eosin (H&E)-stained formalin-fixed, paraffin-embedded (FFPE) tumor tissue samples. We provided a proof-of-concept prediction of the Ki67/MIB1 IHC positivity of cancer cells through the definition and quantitation of single nuclear features. In the first instance, we set our digital framework on Ki67/MIB1-stained OSCC (oral squamous cell carcinoma) tissue sample whole slide images, using QuPath as a working platform and its integrated algorithms, and we built a classifier in order to distinguish tumor and stroma classes and, within them, Ki67-positive and Ki67-negative cells; then, we sorted the morphometric features of tumor cells related to their Ki67 IHC status. Among the evaluated features, nuclear hematoxylin mean optical density (NHMOD) presented as the best one to distinguish Ki67/MIB1 positive from negative cells. We confirmed our findings in a single-cell level analysis of H&E staining on Ki67-immunostained/H&E-decolored tissue samples. Finally, we tested our digital framework on a case series of oral squamous cell carcinomas (OSCC), arranged in tissue microarrays; we selected two consecutive sections of each OSCC FFPE TMA (tissue microarray) block, respectively stained with H&E and immuno-stained for Ki67/MIB1. We automatically detected tumor cells in H&E slides and generated a “false color map” (FCM) based on NHMOD through the QuPath measurements map tool. FCM nearly coincided with the actual immunohistochemical result, allowing the prediction of Ki67/MIB1 positive cells in a direct visual fashion. Our proposed approach provides the pathologist with a fast method of identifying the proliferating compartment of the tumor through a quantitative assessment of the nuclear features on H&E slides, readily appreciable by visual inspection. Although this technique needs to be fine-tuned and tested on larger series of tumors, the digital analysis approach appears to be a promising tool to quickly forecast the tumor’s proliferation fraction directly on routinely H&E-stained digital sections.

## 1. Introduction

The assessment of the replicative activity of the cells and their ability to proliferate, or specifically the frequency they enter into the mitotic phase of the cell cycle, are major determinants of the biologic behavior of several human tumors. To this aim, one of the most used tools in surgical pathology is the IHC (immunohistochemical) labeling index (LI) of the Ki67 nuclear protein, assessed by immunostaining with the MIB1 monoclonal antibody on FFPE (formalin-fixed, paraffin-embedded) tissue sections [1,2]. Ki67 antigen was initially identified in the early 1980s by Scholzer and Gerdes and encodes for two isoforms of 345kDa and 395kDa [3]. Ki67 protein expression depends on the proliferative activity of cells, is expressed in all the cell cycle phases but G0, and can be used as an aggressiveness biomarker of malignant tumors [4,5]; therefore, pathologists routinely employ the Ki-67 labeling index as a proliferation marker [6].

The protein Ki67 has been suggested as a diagnostic biomarker in several tumors, being overexpressed in malignant tumor tissues compared to normal ones [7,8], and it correlates to tissue differentiation in an inversely proportional fashion; many studies have shown a correlation between the Ki67/MIB-1 labeling index and human cancer grading [4,9,10,11,12,13,14]. Moreover, it correlates with the clinical tumors’ stage and occult metastasis [15,16,17,18], and Ki67 expression evaluation, in combination with other histopathological characteristics, may also represent an indicator of the risk of tumor recurrence [19].

The prognostic value of Ki67 IHC labeling has been demonstrated in several human solid tumors such as breast, soft tissue, lung, prostate, cervix, and central nervous system [20,21,22,23,24].

Different approaches have been proposed so far to optimize the Ki67 LI evaluation through digital image analysis of Ki67 IHC-stained glass slides, but none of them are about the Ki67 IHC positivity prediction from an H&E (hematoxylin and eosin)-stained glass slide [25,26].

Nowadays, most of the routine practice in pathology facilities typically relies on the assessment of small biopsies. In this framework, the reduction in biospecimen consumption for each analysis is mandatory to save material for special staining or molecular biology examination.

For this reason, we explored the possibility of predicting Ki67 labeling using hematoxylin and eosin (H&E)-stained digitalized histological sections, by uncovering morphological and densitometric features that could distinguish between proliferative and quiescent neoplastic cells, such as nucleus area and perimeter, that reflect the increase in dimension of the nuclei, and hematoxylin optical density, that reflects chromatin condensation. We then developed a new algorithm that may be applied to different tumors to evaluate the proliferative tumor cells fraction, using QuPath [27], an open-source software. In the first instance, we analyzed a case series of OSCC (oral squamous cell carcinoma) H&E-stained digital slides using a digital pathology approach. We used QuPath to manually annotate different tumoral and stromal area on TMA (tissue microarrays) to segment nuclei, in order to create our dataset and generate different classifiers using the QuPath "Object Classification" function. In this pilot study, we explored how machine-learning on H&E-based morphometric features could distinguish the proliferation-committed fraction of neoplastic cells (which immunohistochemistry detects by Ki67-positive nuclear stain) in OSCC, to allow pathologists to evaluate this fundamental feature (information) directly on H&E-stained FFPE tumor slides (Figure S1).

As far as we know, this is the first time QuPath has been used to predict the H&E equivalent of an immunohistochemical positivity and the first description of the application of computational pathology to predict OSCC biomarkers’ expression on routinely stained H&E tumor sections.

## 2. Results

### 2.1. Ki67-Positive Cells Show a Higher Nuclear Mean Hematoxylin Optical Density

In the first instance, we intended to extract, as described in the Materials and Methods, on immunostained whole slide images, a series of morphological and densitometric features that could distinguish Ki67-positive cells from Ki67-negative ones. 

We firstly sorted tumoral and stromal cells using a Random Trees classifier generated with QuPath embedded tools, as described in the Materials and Methods. Table 1 summarizes the performances of all tested classifiers. 

Employing the chosen classifier, we automatically identified stromal and tumoral cells classes. Within the latter, we identified Ki67-positive and Ki67-negative cells based on the estimated staining vector set in the preprocessing phase and included in the trained classifier, using QuPath "intensity feature" tool. 

**Figure 1:**
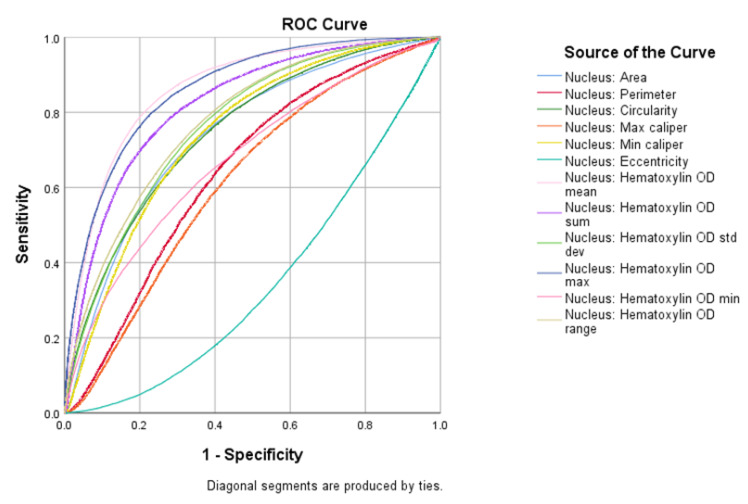
Plot of all nuclear feature ROC curves. NHMOD is the best feature to tell positive from negative cells.

Following the cell detection phase among all the QuPath computed features, we extracted the ones which referred to nuclear characteristics and evaluated the ability of each to classify Ki67-positive and negative cells. The ROC (Receiver Operating Characteristic) curve analysis revealed that nuclear hematoxylin mean optical density (NHMOD) was the nuclear feature with the best performance (Figure 1).

### 2.2. Validation on H&E-Decolored/Ki67 Immuno-Stained Slides

We validated NHMOD’s ability to distinguish Ki67-positive tumor cells on OSCC H&E-stained slides that, following digitalization, were decolored and subsequently IHC-stained with an anti-Ki67 antibody.

**Figure 2:**
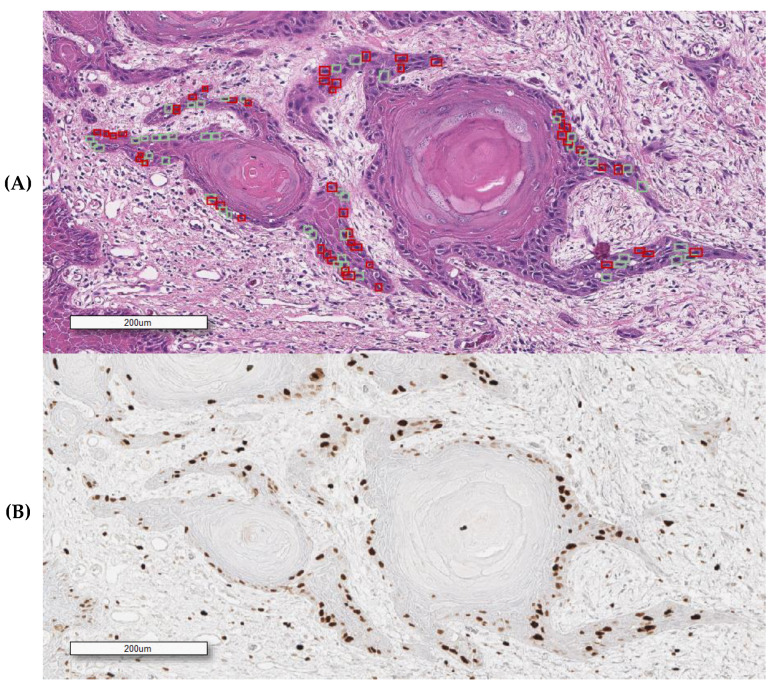
An overlapping field with SCC (squamous cell carcinoma) obtained from the Ki67 immunostained H&E decolored sample. (**A**) H&E (hematoxylin and eosin) (squares indicate some of the selected cells for feature analysis); (**B**) Ki67/MIB1 IHC (immunohistochemical). Red and green squares, respectively, represent positive and negatively annotated cells.

**Figure 3:**
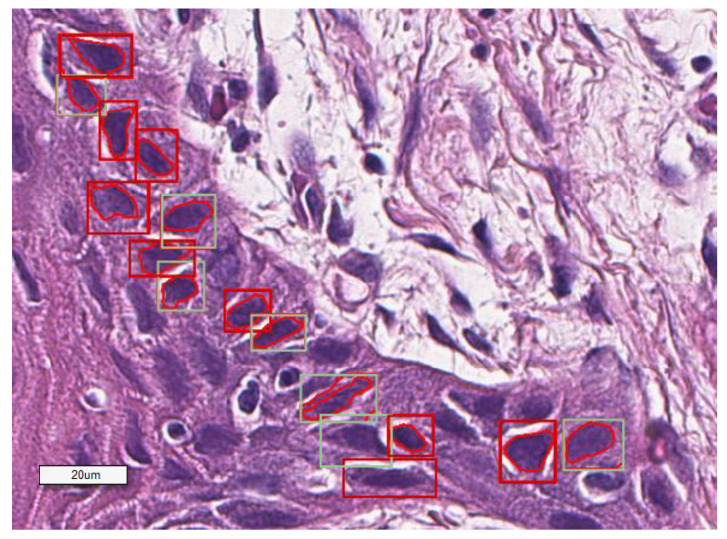
A representative image showing how the software classifies the selected cells based on NHMOD.

**Figure 4:**
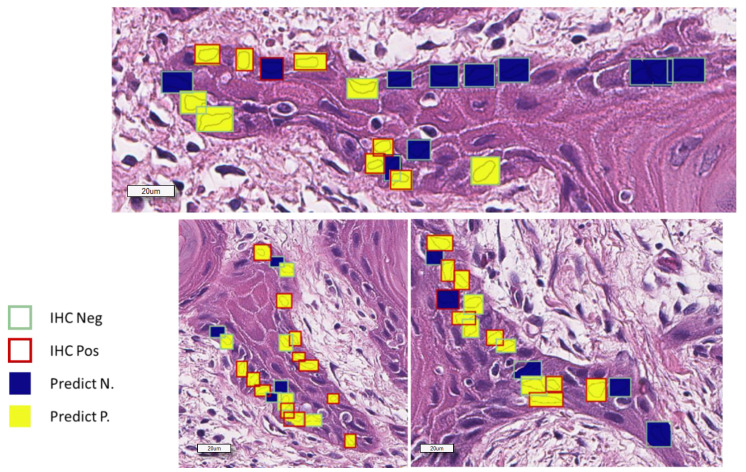
Representative image showing the graphical report of Ki67 status prediction. The overlapped squares color shows the real Ki67 status as observed in the corresponding field of Ki67 immunostained section.

We manually annotated Ki67-positive and negative tumor cells on H&E slides using the corresponding Ki67 IHC-stained one as ground truth, aligning the H&E field of view with the corresponding IHC one (Figure 2). For our analysis, we tested our algorithm on H&E-stained slides generated by different laboratories to ensure the reproducibility of the data. This approach allowed us to analyze the efficiency of the morphometric-based prediction of Ki67 positivity at a single-cell level on the same tissue section stained with different H&E (Figure 3 and Figure 4).

We compared all the nuclear features, extracted from the H&E stain, of the Ki67-positive and Ki67-negative tumor cells, and we confirmed a statistically significant difference only for nuclear hematoxylin mean optical density. We confirmed, on a single-cell level analysis, that NHMOD is the morphometric feature that better distinguishes the Ki67-positive cell population from the Ki67-negative one (AUC = 0.688, 95% C.I: 0.639–0.740, F1-Score = 0.638). 

The statistic values of our test are summarized in Table 2 and Table 3.

### 2.3. Morphometry-Based False Color Map Matches with Ki67 IHC Expression Pattern

**Figure 5:**
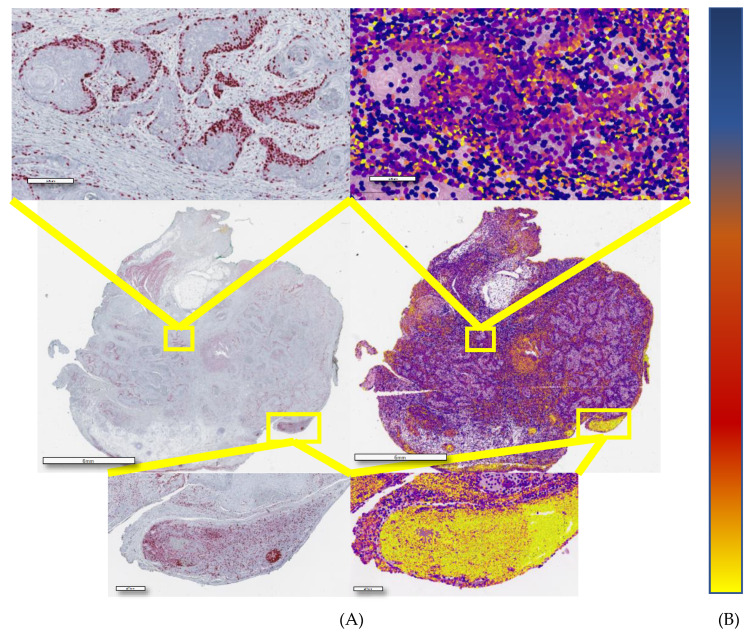
(**A**) Immunostaining and the corresponding NHMOD false color map (**B**) Colourmap from the lowest (top) to the highest (bottom) probability of positivity.

Following the selection of the best sorting feature, we applied the same algorithm on tissue microarray (TMA) H&E slides to generate false color maps (FCMs), with a color scale ranging from blue to yellow, where blue corresponds to the lowest value of the observed feature and yellow is the highest one. The nuclear hematoxylin mean optical density (NHMOD)-based map nearly coincided with the actual immunohistochemical slide results (Figure 5), allowing the pathologist to visually and very easily envisage the Ki67 positivity state. We considered a core positive to Ki-67 if more than 10% of cells were red or more (according to color-ramp in Figure 4). The estimated sensitivity of the proposed visual prediction is 100%, the estimated specificity being about 55% compared to actual Ki67 positivity.

The proposed visual approach may give the pathologist a fast and straightforward method to identify the proliferating compartment of the tumor, testing only positive-resulted cases to confirm the result.

## 3. Discussion

Ki67 is a reliable marker of tumor proliferation and correlates with prognosis, progression, and metastatic risk in different human tumors [28,29]. Despite its well-known role in several neoplasias, there is still a lack of confirmation for a possible diagnostic, prognostic, or predictive role in some others, like in oral squamous carcinoma (OSCC).

Head and neck cancer (HNC) is the eighth most common malignancy in the world, with squamous cell carcinoma (SCC) accounting for more than 90% and being the most frequent form of OSCC [30]. Head and Neck Squamous Cell Carcinomas (HNSCCs) are characterized by a high rate of morbidity and mortality. Furthermore, in most countries, the five year survival rate is less than 50% [31], and, in the United States, more than 20,000 new cases are estimated to occur in 2019, with more than 10,000 deaths [30].

Results on the correlation between Ki-67 expression and individual oral cancer patient prognosis are highly controversial and the prognostic role of Ki67 is still a matter of debate among the scientific community [32]. Ki67 labeling index has shown no reproducible prognostic value in OSCC. In particular, its contribution in determining tumor fate usually emerged when the immunohistochemical evaluation also involved other cell cycle proteins [33]. This finding, concerning the well-defined role of the Ki67 labeling index in the primary diagnosis and risk stratification of most human solid malignant tumors, is contradictory [34], and further investigation is necessary.

To date, the vast majority of routine practice in surgical pathology relies on histopathological examination of small biopsies. The pressure for earlier diagnosis and the necessity to reduce the invasiveness of diagnostic histopathology sampling procedures result in the samples being analyzed becoming smaller and smaller, and very often biospecimen consumption is reduced to save them for eventual special staining, or molecular biology examination is mandatory. Our study demonstrates the feasibility of predicting Ki67 status in oral squamous carcinoma using hematoxylin and eosin-stained histopathology glass slides and QuPath, an open-source histopathology image analysis software, allowing the collection of information about proliferation status on routine basic staining.

Our machine-learning based algorithm focused on selected nuclear features able to distinguish between Ki67-positive and Ki67-negative immunostained tumor cells. Our interest in nuclear morphometric features was initially oriented by experience, since nuclear morphology is a leading morphological focus point in the routine histopathological diagnosis of human solid tumors; moreover, since we were looking at the proliferating fraction of the studied tumor samples, nuclear shape and morphometrical determination of chromatin compaction were the features we looked at in the first instance. We were not surprised to find out that nuclear hematoxylin optical density turned out to be significantly associated with Ki67 IHC positivity, since it may reflect the attitude of the nuclear chromatin to predispose itself for DNA replication.

We obtained a significant result by decoloring H&E-stained tumor sections and then immunostaining them with an anti-Ki67/MIB1 antibody. Only by this approach could we obtain a single-cell level analysis to confirm our findings. Nevertheless, we are aware that IHC staining on decolored sections could underestimate the real Ki67 positivity in the tumor, for mere technical reasons, and we are confident of reducing the number of false-negative results of our test by further improving the IHC protocol following decoloration.

In our single-cell analysis, we choose the cells to analyze by sorting them from invasive tumor nests or the invasive tumor’s front, i.e., the tumor’s regions expected to proliferate the most, since we know that this is the most critical to evaluate concerning the proliferative potential. In the most differentiated and less mitotically active region of the tumor, the performance of our test proved to be even better.

We envisage, in the near future, a prognostic role of morphometric features; we believe in the possibility to stratify the risk of poor outcome of OSCC based only on the estimation of morphometric features via AI-based algorithm.

We propose a visual inspection system based on a false colored map of histopathology samples, generated following NHMOD quantitation, and converting NHMOD values to an easy to interpret color scale.

This approach may help pathologists in their diagnostic procedure, forecasting the evaluation of the proliferative fraction of solid tumors, based on a Ki67/MIB1 IHC positivity prediction on H&E-stained glass slides. The application of this algorithm to the routine diagnostic could provide the pathologist the chance to limit the request of a KI67/MIB1 immunohistochemical stain, restricting its use to when case results predict as positive alone, in order to reduce costs and turn-around time. This algorithm may also be useful in telepathology, providing emerging countries or smaller centers the possibility to have more information about the proliferative status of a lesion without the necessity of an immunohistochemical stain. In order to translate our proof of concept into a useful tool for computer-aided diagnostics, we believe in the full integration of AI-based image analysis algorithms in anatomic pathology units’ management software.

Furthermore, to our knowledge, there are few works using machine learning or deep learning to predict immunohistochemical expression [35,36] or mutational status [37], but this is the first that provides information about Ki67 levels on hematoxylin/eosin stains using an open-source software with a user-friendly interface. This aspect is particularly important, because it is possible to process whole slide images even with modest hardware; all the image analyses were performed with an Acer Aspire E15 with 16GB RAM, 128GB SSD, and an NVIDIA GeForce 940MX (NVIDIA, Santa Clara, CA, USA), which are easily affordable.

Although a promising result, our study still presents some limitations. We tested our algorithm on TMAs, so it will be necessary to verify its performance on whole slide images that may differ from TMAs.

Future steps in the development of this algorithm will be on slides from even more laboratories and different types of lesions. Then, a more accurate analysis of larger datasets is necessary to increase the sensibility and sensitivity of this technique, to develop a color normalization tool and to define a standard threshold to define Ki67-positivity.

## 4. Materials and Methods

### 4.1. Dataset Acquisition

We retrieved the FFPE case study population from the Pathology Unit’s archive of the University of Naples “Federico II.” OSCC FFPE tumor samples, from surgical resections, were used to build tissue microarrays (TMAs). Three TMAs were built containing 111 tumor samples, selecting the most representative areas from each selected paraffin block, at least in duplicate. Then, 3 mm tissue cores were punched from morphologically representative tissue areas of each donor block and placed into one recipient paraffin block (3 × 2.5 cm) using a semi-automated tissue arrayer (Galileo TMA, Milan, Italy). One section of each TMA (4 μm) was stained with hematoxylin and eosin (H&E) to check the adequacy of cores.

H&E-stained and IHC-stained glass slides were digitalized at 40× using the Leica Aperio AT2 slide scanner (Leica Biosystems, Vista, CA, USA).

The study was performed according to the guidelines of the institutional ethics committee, which, in agreement with Italian law, concerning the topics of the current research, and according to Declaration of Helsinki, requires, for studies based only on retrospective analyses on routine archival FFPE-tissue, a written informed consent from the living patients, following the indication of Italian DLgs No. 196/03 (Codex on Privacy), as modified by the UE 2016/679 law of the European Parliament and Commission at the time of surgery.

### 4.2. Destaining

For this study, we selected paraffin-embedded tissue blocks of OSCC, retrieved from the archives of the Pathology Section of the Department of Advanced Biomedical Sciences, ‘Federico II’ University of Naples. Ordinary 4-µm tissue sections were cut from the paraffin blocks on charged slides to avoid detaching tissue sections from the slides. The slides were stained routinely with hematoxylin and eosin and digitized using an Aperio AT2 scanning system using 20× objective with a 2× multiplier inserted, obtaining 40× WSI.

After slide scanning, the coverslips were removed by soaking the slides in xylene until the coverslips detached. The slides were rehydrated in decreasing concentrations of ethanol and then destained using a solution of HCl 0.3% for 4 min.

After destaining, the slides were rinsed in tap water and subsequently immunostained with the antibody anti-Ki67. Immunohistochemical staining was performed on a Ventana Benchmark Ultra (Ventana Medical Systems Inc., Tucson, AZ, USA) using the rabbit monoclonal antibody anti-Ki67 (clone 30-9, Ventana Medical Systems Inc.) in accordance with both manufacturers’ recommendations. The new IHC-stained slides were then digitized.

### 4.3. Immunohistochemistry

For the Ki67 IHC assay, fully automated staining was performed on a Ventana Benchmark Ultra (Ventana Medical Systems Inc.) using the primary antibody anti-Ki-67 (rabbit monoclonal, clone 30–09 Ventana Medical Systems, Inc.), according to manufacturers’ recommendations. The sections were incubated with primary antibodies and revealed with Ultra View Universal Alkaline Phosphatase Red Detection Kit (Ventana Medical Systems Inc.).

### 4.4. Annotation and Processing

After H&E and IHC staining, slides were scanned with Leica AperioAT2 at 40×. The slides were analyzed using QuPath, an open-source software which allows us to perform WSI analysis through tissue and nuclei segmentation and to automatically compute a series of features with various algorithms. All the calculated features are listed in Table S1. We firstly identified stain vectors using QuPath tools, then we dearrayed our TMAs and used the "Tissue Detection" to define the ROI containing tissue within the core. After a step of evaluable core selection, excluding all non-assessable cores and all cores without tumors, we used the "Cell Detection" tool to segment nuclei. A pathologist then manually annotated different tumoral and stromal areas on the first TMA using QuPath to create our dataset and generated different classifiers using QuPath "Object Classification". The dataset is composed of 6065 objects (nuclei), using a validation split of 0.2. Among the generated classifiers (Random Trees, Normal Bayes Classifier, SVM and K-nearest, using QuPath default parameters), we decided to apply the Random Trees classifier to identify the tumor and non-tumor cells and calculate cell features, as it presented as the most accurate. Random tree parameters were left as default (maximum tree depth: unlimited; minimum samples per node: 10; termination criterion-max trees: 50; termination criterion accuracy: disabled)

We found that nuclear hematoxylin mean optical density was the most valuable feature in distinguishing positive from negative cells, so we applied the same algorithm on H&E TMA-stained glasses and generated a false color map.

### 4.5. Data Analysis and Statistics

Single-cell features were exported from QuPath and analyzed using SPSS (IBM Corp. Released 2013. IBM SPSS Statistiimcs for Windows, Version 22.0. Armonk, NY, USA).

## 5. Conclusions

In conclusion, although the proposed technique needs to be fine-tuned and tested on larger series of tumors in different staining conditions, the digital analysis approach appears to be a promising tool to quickly forecast the tumor’s proliferation fraction on simple H&E-stained digital sections.

## Figures and Tables

**Table 1:** Performance of different tested classifiers using the same training and validation sets for each classifier.

Classifier	Accuracy	95% CI:
Random Trees	98.19%	97.27 to 98.86%
SVM	98.02%	97.07 to 98.73%
K-Nearest	73.04%	69.77 to 74.88%
Normal Bayes Classifier	69.25%	66.57 to 71.84%

**Table 2:** Statistics of NHMOD (nuclear hematoxylin mean optical density) as a key feature in distinguishing Ki67-positive from Ki67-negative cells.

Statistic	Value	CI 95%
Specificity	69.05%	62.32 to 75.23%
Sensitivity	62.83%	55.55 to 69.69%
Negative Predictive Value	67.13%	62.45 to 71.49%
Positive Predictive Value	64.86%	59.47 to 69.90%
Accuracy	66.08%	61.22 to 70.71%

**Table 3:** Confusion Matrix of Ki67-predicted positive or negative from Ki67-annotated ground truth.

		Prediction		Total
		Positive	Negative	
**Ground Truth**	**Positive**	120	71	210
	**Negative**	65	145	191
	**Total**	216	185	401

## Supplementary Materials

The following are available online at https://www.mdpi.com/2072-6694/12/5/1344/s1, Figure S1: Flow chart of the study’s design, Table S1: List of QuPath computed nuclear features (OD: Optical Density).
