# Supplementary material for: A Machine-learning Approach for the Assessment of the Proliferative Compartment of Solid Tumors on Hematoxylin-Eosin-Stained Sections
Source: Cancers (Basel). 2020 May 25;12(5):1344. doi: 10.3390/cancers12051344 (PMC7281627; doi:10.3390/cancers12051344)
Supplement: Supplementary file 1 [file cancers-12-01344-s001.pdf]

cancers

MDPI

A Machine-Learning Approach for the Assessment of the Proliferative Compartment of Solid Tumors on Hematoxylin-Eosin Stained Sections

Francesco Martino, Silvia Varricchio, Daniela Russo, Francesco Merolla, Gennaro Ilardi, Massimo Mascolo, Giovanni Orabona dell'Aversana, Luigi Califano, Guglielmo Toscano, Giuseppe De Pietro, Maria Frucci, Nadia Brancati, Filippo Fraggetta and Stefania Staibano

SupplementaryMaterials

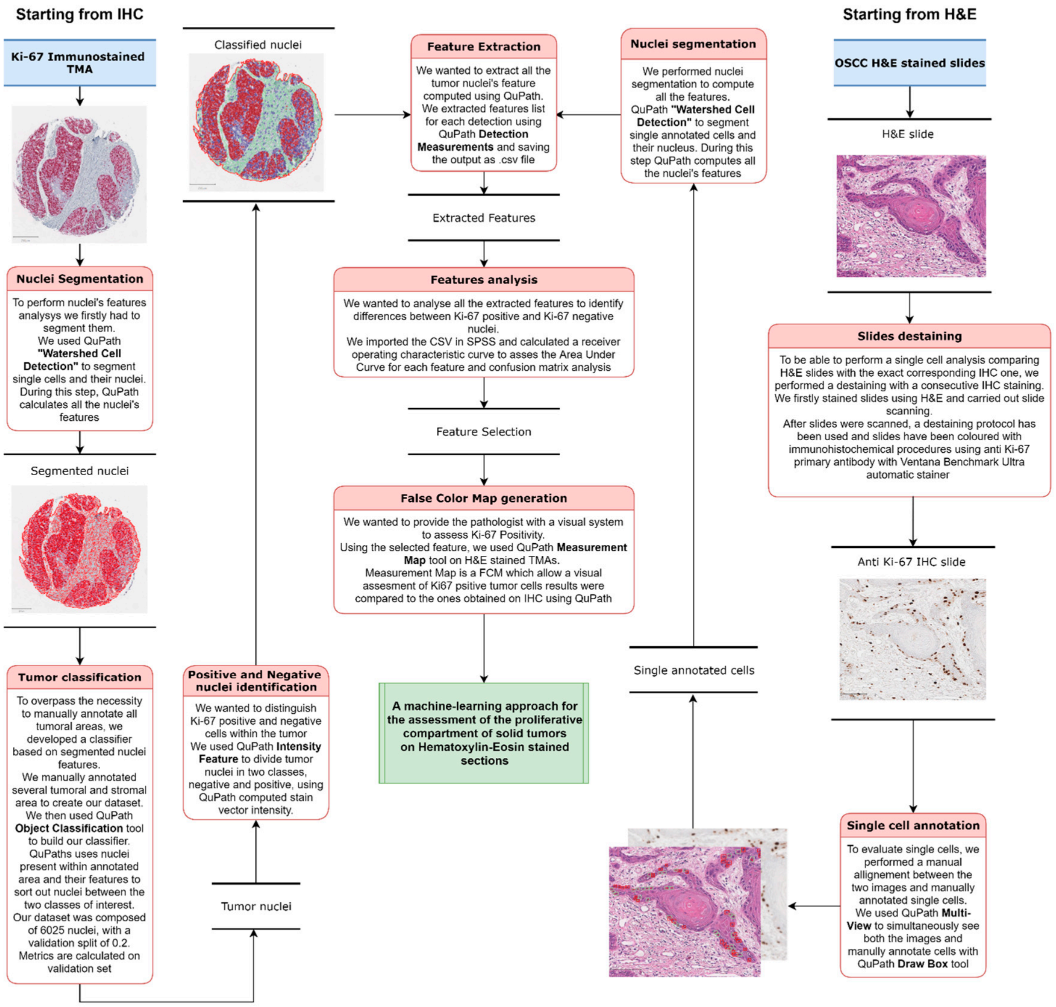

Figure S1. Flow chart of the study's design.

Table S1. List of QuPath computed nuclear features (OD: Optical Density).

Compartment	Features
Nucleus:	Area<br>Perimeter<br>Circularity<br>Max caliper<br>Min caliper<br>Eccentricity<br>Hematoxylin OD mean<br>Hematoxylin OD sum<br>Hematoxylin OD std dev<br>Hematoxylin OD max<br>Hematoxylin OD min<br>Hematoxylin OD range<br>Stain Vector OD mean<br>Stain Vector OD sum<br>Stain Vector OD std dev<br>Stain Vector OD max<br>Stain Vector OD min<br>Stain Vector OD range
Cell:	Area<br>Perimeter<br>Circularity<br>Max caliper<br>Min caliper<br>Eccentricity<br>Hematoxylin OD mean<br>Hematoxylin OD sum<br>Hematoxylin OD std dev<br>Hematoxylin OD max<br>Hematoxylin OD min<br>Hematoxylin OD range<br>Stain Vector OD mean<br>Stain Vector OD sum<br>Stain Vector OD std dev<br>Stain Vector OD max<br>Stain Vector OD min<br>Stain Vector OD range
Cytoplasm:	Area<br>Perimeter<br>Circularity<br>Max caliper<br>Min caliper<br>Eccentricity<br>Hematoxylin OD mean<br>Hematoxylin OD sum<br>Hematoxylin OD std dev<br>Hematoxylin OD max<br>Hematoxylin OD min<br>Hematoxylin OD range<br>Stain Vector OD mean<br>Stain Vector OD sum<br>Stain Vector OD std dev<br>Stain Vector OD max<br>Stain Vector OD min<br>Stain Vector OD range
